# Antiproliferative and apoptotic effects of telmisartan in human glioma cells

**DOI:** 10.1186/s12935-023-02963-1

**Published:** 2023-06-09

**Authors:** Yung-Lung Chang, Chung-Hsing Chou, Yao-Feng Li, Li-Chun Huang, Ying Kao, Dueng-Yuan Hueng, Chia-Kuang Tsai

**Affiliations:** 1 Department of Biochemistry, National Defense Medical Center, Taipei, 11490 Taiwan; 2 Department of Neurology, Tri-Service General Hospital, National Defense Medical Center, No. 325, Sec. 2, Cheng-Gong Road, Taipei, 11490 Taiwan; 3 Department of Pathology, Tri-Service General Hospital, National Defense Medical Center, Taipei, 11490 Taiwan; 4 Division of Neurosurgery, Department of Surgery, Taipei City Hospital Zhongxing Branch, Taipei, Taiwan; 5 Department of Neurological Surgery, Tri-Service General Hospital, National Defense Medical Center, Taipei, 11490 Taiwan

**Keywords:** Telmisartan, Glioblastoma, SOX9, Proliferation, Apoptosis

## Abstract

Glioblastoma is the most common primary central nervous system tumor in adults. Angiotensin II receptor blockers (ARBs) are broadly applied to treat hypertension. Moreover, research has revealed that ARBs have the capacity to suppress the growth of several cancer types. In this study, we assessed the effects of three ARBs with the ability to cross the blood brain barrier (telmisartan, valsartan and fimasartan) on cell proliferation in three glioblastoma multiforme (GBM) cell lines. Telmisartan markedly suppressed the proliferation, migration, and invasion of these three GBM cell lines. Microarray data analysis revealed that telmisartan regulates DNA replication, mismatch repair, and the cell cycle pathway in GBM cells. Furthermore, telmisartan induced G0/G1 phase arrest and apoptosis. The bioinformatic analysis and western blotting results provide evidence that SOX9 is a downstream target of telmisartan. Telmisartan also suppressed tumor growth in vivo in an orthotopic transplant mouse model. Therefore, telmisartan is a potential treatment for human GBM.

**Supplementary Information:**

The online version contains supplementary material available at 10.1186/s12935-023-02963-1.

## Introduction

Brain cancer accounts for only 2% of cancers, but it is notoriously difficult to treat. Intracranial brain tumors can be categorized as primary and secondary tumors, and the most common primary central nervous system (CNS) tumor in adults is glioma, which accounts for 29% of primary CNS tumors [1, 2]. Survival time is affected by histology; pilocytic astrocytoma has a 10-year survival of more than 90%, while GBM has a 5-year survival rate of only 5% [3, 4]. Overall, the median survival time of GBM patients is 12 months after standard surgical resection and postoperative radiotherapy; if the oral chemotherapy drug temozolomide is added to the regimen, the median survival is only extended to 16 months [3, 4]. Therefore, there is an urgent need to develop adjuvant treatments for GBM.

The renin-angiotensin system (RAS) plays an important role in the homeostasis of different tissues. Angiotensin II (Ang II) is an octapeptide hormone that has crucial biological effects on features and processes including cardiovascular volume, blood pressure control, water and salt balance, and neuroendocrine function [5, 6]. Current studies have revealed that the biological effect of Ang II is mediated through two G protein-coupled receptors, angiotensin II receptor type I (AT1R, encoded by *AGTR1*) and angiotensin II receptor type II (AT2R, encoded by *AGTR2*), which are expressed in normal and various cancer cells, including cancer cells of the ovary, prostate, pancreas, breast and gut [7]. Most related reports have focused on AT1R expression (and, to a lesser extent, AT2R expression). Briefly, the activation of *AGTR1* triggers numerous signaling cascades critical for the downstream regulation of angiogenesis, vessel remodeling, cell growth, inflammation and fibrosis [9]. Since *AGTR1* plays an important role in cancer progression, the knockdown of AT1R could potentially be an advantageous complementary treatment strategy. Moreover, abnormal RAS constituent expression may induce tissue-specific malignant transformation [7].

There are limited studies on RAS expression and glioma. Juillerat-Jeanneret et al. investigated the role of RAS in the growth and apoptosis of human glioblastoma. Nevertheless, they found that ACE inhibitors did not reduce glioblastoma cell proliferation [8]. Another study by Arrieta et al. reported that the high angiotensin II receptor expression is correlated with a poor prognosis, as proven by clinical data, but they did not perform a further in vitro mechanism survey [9]. Another study described that tumor growth of cultured C6 rat glioma cells was inhibited by losartan (an ARB) at doses of 40 or 80 mg kg^−1^ [10].

The present study aims to find a new treatment strategy for glioma. Repurposing of old drugs is a valuable research strategy. We initially selected three ARBs, telmisartan, valsartan, and fimasartan, for preliminary study. All three ARBs can cross the blood brain barrier, as evidenced by previous animal studies [11–14]. The results demonstrate that telmisartan can reduce glioma cell growth. Telmisartan also inhibits GBM cell migration and invasion. Finally, we discovered via *in vivo* experiments that SOX9 is a potential downstream target and induces a therapeutic effect.

## Materials and methods

### Chemicals

The following chemicals were utilized: telmisartan (A10905, AdooQ), valsartan (SML0142, Sigma), and fimasartan (HY-B0780, MedChemExpress).

### Cell culture and transfection

GBM cells were cultured in Dulbecco’s Modified Eagle Medium (DMEM) (U87MG, LNZ308: 10% FBS, LN229: 2% FBS) supplemented with 2% fetal bovine serum, penicillin, and streptomycin at 37 °C under 5% CO2 and 95% air, as formerly depicted in Tsai et al. [15].

### Colony formation assay

Glioma cells were seeded out in 6-well plates at a density of 1000 cells per well and cultured for 2 weeks. The colonies were then fixed with 100% methanol for 10 min at room temperature and stained with 0.005% crystal violet for 10 min. Colonies larger than 0.5 mm in diameter were counted by ImageJ software (National Institutes of Health, Bethesda, MD, USA).

### MTS cell viability assay

Cell viability assays were carried out as described previously [16]. We used MTS assays with Cell Titer 96Aqueous One Solution Reagent (Promega, Madison, WI, USA) to assess cell viability. Briefly, LN229, U87MG, and LNZ308 cells treated with indicated drugs for 24, 48, and 72 h were respectively incubated at a density of 10^3^ cells per well for 2 h in DMEM (200 ml) supplemented with MTS solution (20 μl/well), after which the absorbance at 490 nm was recorded using a Varioskan™ LUX multimode microplate reader (Thermo Fisher Scientific). Three independent experiments were performed.

### Flow cytometry

We used FITC bromo-deoxyuridine (BrdU) flow kits (#559619, BD Biosciences) to evaluate GBM cell proliferation according to the manufacturer’s instructions. In brief, 3 × 10^5^ of LN229, U87MG, and LNZ308 cells were seeded in six-cm plates and incubated overnight. Afterwards, cells were treated with indicated concentrations of telmisartan for 48 h. The cells were then harvested, labeled with BrdU for 1 h, washed and treated with DNAase at 37 °C for 1 h, followed by labeling with FITC anti-BrdU for 20 min and 7-AAD for 20 min at room temperature in the dark. The percentage of positive glioma cells was determined using flow cytometry (BD Biosciences).

We utilized the PE Annexin V Apoptosis Detection Kit (#559763, BD Biosciences) to detect apoptosis. In brief, 3 × 10^5^ of LN229, U87MG, and LNZ308 cells were seeded respectively in six-cm plates and incubated overnight. Afterwards, cells were treated with indicated concentrations of telmisartan for 48 h. The cells were then harvested, rinsed, and labeled with PE Annexin V and 7-AAD according to the manufacturer’s protocols. Stained cells were assessed by flow cytometry (BD Biosciences) after treatment.

For cell cycle analysis, 3 × 10^5^ of LN229, U87MG, and LNZ308 cells were seeded respectively in six-cm plates and incubated overnight. Afterwards, cells were treated with indicated concentrations of telmisartan for 48 h. The cells were then washed twice with cold phosphate-buffered saline and stained with propidium iodide (PI) (#1932759, Invitrogen) for 30 min in the dark. Cell-cycle analysis was performed by evaluating DNA content using fluorescence activated cell sorting (BD Biosciences). Three independent experiments were performed.

### Western blot analysis

LN229, U87MG, and LNZ308 cells were seeded respectively in 10-cm plates and incubated with indicated concentrations of telmisartan for 48 h. The cells were washed in PBS and then lysed with RIPA lysis buffer. Cell lysates were separated by sodium dodecyl sulfate (SDS)-polyacrylamide gel electrophoresis (PAGE), followed by transferring to a polyvinylidene difluoride membrane (Millipore, Bedford, MA, USA). The membrane was blocked with 5% skim milk, and then incubated with the primary antibody. These membranes were then incubated with appropriate secondary antibody. Bands were visualized by an enhanced chemiluminescence (ECL) Detection Reagent (Cytiva, Little Chalfont, Buckinghamshire UK).

Western blot analyses were performed using cyclin B1 (#4135, Cell Signaling Technology), CDK1 (#77055, Cell Signaling Technology), CDK2 (#2546, Cell Signaling Technology), and SOX9 (#82630, Cell Signaling Technology) antibodies. Anti-β-actin (sc-47778, Santa Cruz Biotechnology), GAPDH (sc-47724, Santa Cruz Biotechnology), and anti-actinin (ACTN; sc-17829, Santa Cruz Biotechnology,) were used to detect endogenous reference genes.

### Global gene expression profiling

Total RNA from LN229 cells was analyzed using Human One Array Plus (Phalanx Biotech Group, Hsinchu, Taiwan). The gene expression values were established by Agilent Technologies (Santa Clara, CA, USA) 0.1 XDR Protocol. Fold changes were determined as the ratio of the mean values for telmisartan-treated cells to the values for the control cells.

### Bioinformatics search of The Cancer Genome Atlas data

To determine the role of *AGTR1* mRNA expression in glioma, we obtained data from The Cancer Genome Atlas (TCGA; UCSC Xena) after sorting. Cases with missing values were excluded from the analysis. The samples acquired from the TCGA_GBMLGG dataset (https://xenabrowser.net/, accessed on 2 March 2022) included samples from 662 cases, including 225 grade II, 272 grade III, and 165 grade IV glioma cases. Statistical comparisons of hazard ratios for factors associated with overall survival, including the mRNA expression of *AGTR1* and other glioma-related genes, age, and sex among glioma patients, were performed utilizing one-way ANOVA. These patients were divided into high and low *AGTR1* expression groups based on the median *AGTR1* expression level.

We analyzed a dataset from the TCGA by the Gene Expression Profiling Interactive Analysis (GEPIA) online tool (http://gepia.cancer-pku.cn/) [17]. GEPIA enables customizable functional analyses of TCGA data; the types of analyses include patient survival analysis, differential expression analyses between cancer and normal samples, and gene correlation analyses. We used the Chinese Glioma Genome Atlas (CGGA) (http://www.cgga.org.cn/index.jsp) to assess the association between *AGTR1* expression and survival time [18].

### Quantitative real-time polymerase chain reaction

The protocols for RNA extraction, cDNA synthesis, and quantitative RT‒PCR were reported in Tsai et al. [15]. Amplification and quantification of cDNA were performed using the StepOne Real-Time PCR System (Thermo Fisher Scientific) according to the manufacturer’s instructions. The PCR primers used were as follows: for angiotensin II type 1 receptor (*AGTR1*), 5′-ATTTAGCACTGGCTGACTTATGC-3′ (forward) and 5′-CAGCGGTATTCCATAGCTGTG-3′ (reverse); for glyceraldehyde 3-phosphate dehydrogenase, 5′-GCACCGTCAAGGCTGAGAAC-3′ (forward) and 5′-ATGGTGGTGAAGACGCCAGT-3′ (reverse). The expression of the gene of interest was normalized to the expression of glyceraldehyde 3-phosphate dehydrogenase.

### SOX9 overexpression

GBM cells were transfected with 1 μg SOX9 overexpression plasmid (Takara Bio, Mountain View, CA, USA) and vector (pcDNA3.1) following the Lipofectamine 3000 Transfection protocol (Thermo Fisher Scientific). Untransfected GBM cells were used as the normal control group.

### C6 glioma orthotopic animal model

All animal procedures were approved by the Institutional Animal Care and Use Committee of the National Defense Medical Center. The C57BL/6 mice were anesthetized and immobilized on a stereotaxic surgical table. After shaving the head, a midline incision was made with a high-speed drill at 1 mm anterior and 3 mm lateral to bregma. A Hamilton syringe was used to slowly inject a total 5 × 10^5^ C6 cell suspension with an infusion speed of 0.5 µl/minute at a depth of 5 mm into the bone surface for 3 min before its removal. Then, the burr hole was covered with sterile bone wax, and the incision wound was closed with sutures.

### MRI imaging

All images were acquired with a Bruker PharmaScan^®^ 7 T MRI Scanner outfitted with a 16-cm bore (Bruker, Germany). T2-weighted imaging (T2WI) was performed with a repetition time (TR) of 4000 ms, an echo time (TE) of 60 ms, a field-of-view (FOV) of 20 × 20 mm, a slice thickness of 1 mm, a 256 × 256 acquisition matrix, and an average of 6.

### Sphere formation assay

We used two primary GBM cell lines for the sphere formation assay. We cultured a total of 5 × 10^4^ GBM#1 and GBM#2 cells in medium containing 10% FBS DMEM/F12 (Corning, NY, USA); we harvest and resuspended the cells in 2% FBS DMEM and added them to an ultralow attachment 6-well plate (Corning, NY, USA) for sphere formation assays. Cells were treated with the indicated concentration of telmisartan and cultured for 14 days. We calculated the total number of gliospheres (> 50 μm) under a microscope.

### Statistical analysis

Data are represented as the mean ± standard deviation. Unpaired Student’s *t*-test was performed to compare mean values between the groups. A p value of < 0.05 was considered to indicate statistical significance.

## Results

### Upregulated AGTR1 is associated with a poor survival outcome in GBM patients and with the progression of GBM.

**Fig. 1:**
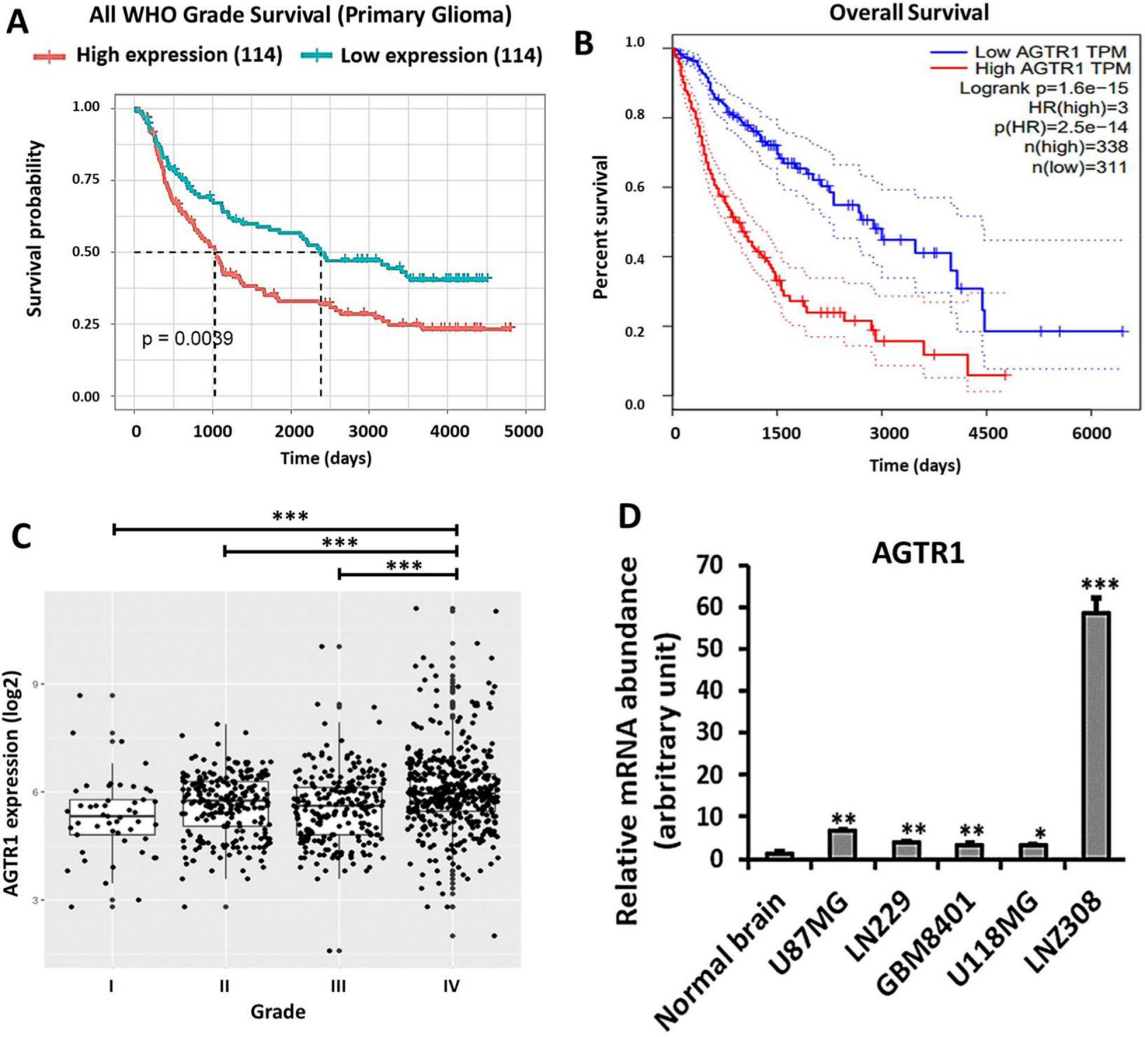
AGTR1 was overexpressed in GBM and correlated with poor survival in glioma patients. **A**, **B** Kaplan‒Meier survival analysis based on *AGTR1* expression in the CGGA and TCGA datasets created using the CGGA and GEPIA platforms, respectively. **C** *AGTR1* mRNA expression in grade I to grade IV glioma is presented as a scatter plot of data obtained from the GENT2 database. Statistical analysis was performed with Student's *t* test (***p < 0.001 compared to the grade IV glioma group). **D** Expression of *AGTR1* mRNA in normal brain, U87MG, LN229, GBM8401, U118MG, and LNZ308 glioma cell lines. Gene expression was normalized to that in the normal brain (*p < 0.05, **p < 0.01, and ***p < 0.001 compared to the normal brain tissue group). These data support that upregulated AGTR1 is associated with a poor survival outcome in GBM patients and with the progression of GBM

To validate *AGTR1* expression and prognosis in GBM patients, we analyzed two datasets, including the CGGA (Fig. 1A) and TCGA (Fig. 1B). High *AGTR1* expression was significantly correlated with shorter survival. In addition, we performed in silico analysis of *AGTR1* expression in human glioma samples of various histological grades from the GENT2 database [19]. *AGTR1* was significantly overexpressed in WHO grade IV GBM compared with WHO grade I, II, and III gliomas (Fig. 1C). We next analyzed *AGTR1* mRNA levels in various glioma cell lines. RT‒PCR analysis revealed that the mRNA expression level of *AGTR1* was significantly higher in glioma cell lines than in normal brain tissue (Fig. 1D).

To assess the relationship between *AGTR1* and glioma-related genes and other factors, we performed multivariate analysis of factors related to overall survival with data for 688 glioma cases from the TCGA dataset. The results revealed that older age (≥ 52 years old), high-grade glioma, and high expression of *AGTR1*, *NF1,* and *AxL* were independent prognostic factors (Table 1). *AGTR1* was considered an indicator of a poor prognosis in GBM patients even after multivariate analysis to eliminate potential confounding effects with other glioma-related genes.

**Table 1:** Multivariate analysis of factors related to overall survival in the TCGA glioma cohort

Multivariate analysis			
Variable	Hazard ratio	95% confidence interval	P Value
Age (≥ 52, < 52)	2.046	1.459–2.870	< 0.001*
PDGFRA expression level (high vs. low)	1.014	0.747–1.376	0.929
Tumor Grade (Gr I, II, III, IV)	2.444	1.875–3.185	< 0.001*
Sex (male vs. female)	0.983	0.751–1.289	0.904
AGTR1 expression level (high vs. low)	1.381	1.020–1.869	0.037*
EGFR expression level (high vs. low)	1.086	0.830–1.421	0.548
TERT expression level (high vs. low)	1.231	0.887–1.709	0.214
ATRX expression level (high vs. low)	0.942	0.709–1.252	0.681
TP53 expression level (high vs. low)	1.096	0.809–1.486	0.553
MGMT expression level (high vs. low)	1.256	0.941–1.677	0.121
NF1 expression level (high vs. low)	0.678	0.478–0.962	0.029*
NF2 expression level (high vs. low)	0.823	0.596–1.137	0.239
NEFL expression level (high vs. low)	0.927	0.713–1.206	0.572
AXL expression level (high vs. low)	1.582	1.214–2.061	0.001*
NR4A1 expression level (high vs. low)	0.860	0.665–1.114	0.253

^*^Indicates statistical significance, *p* < 0.05

### Telmisartan as an effective anticancer agent for glioma

**Fig. 2:**
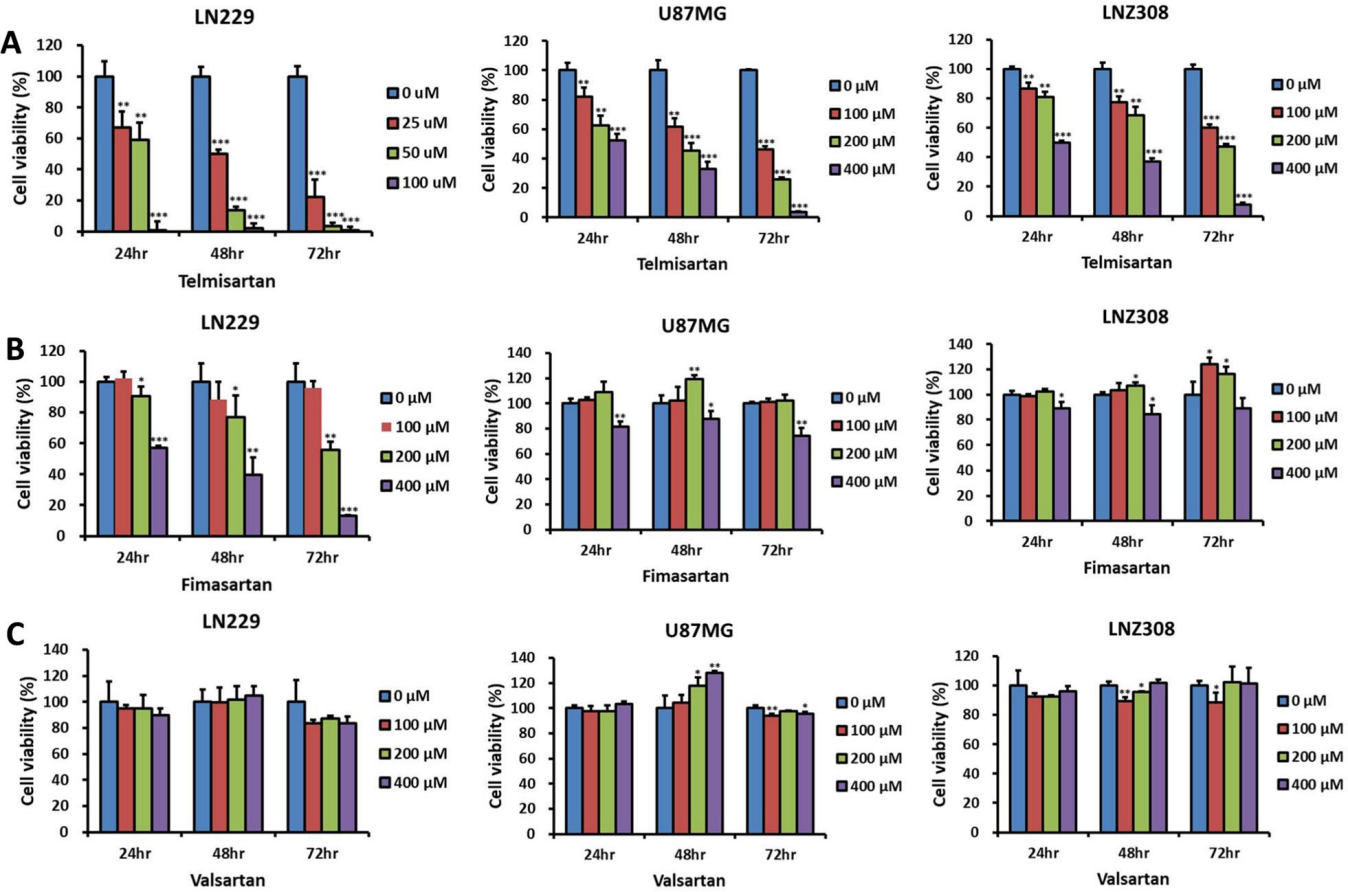
Effects of ARBs on glioma cell viability. Glioma cells (LN229, U87MG, and LNZ308) were treated with telmisartan (**A**), valsartan (**B**), or fimasartan (**C**) at the indicated concentrations or with vehicle control (DMSO), and cell viability was analyzed using the MTS assay at the indicated time points. Values are expressed relative to those of the control group (n = 3, error bars indicate ± SD). Statistical significance was determined using one-way ANOVA followed by Dunnett's multiple comparison test (*p < 0.05; **p < 0.001; ***p < 0.001). These results showed that only telmisartan decreased the viability of all three glioma cell lines in a dose- and time-dependent manner

The AGTR1 gene encodes the angiotensin II type 1 receptor, which is known to mediate the major cardiovascular effects of angiotensin II. We investigated the anticancer effects of angiotensin II type 1 receptor blockers (ARBs) on glioma cells by testing telmisartan, valsartan, and fimasartan on different glioma cell lines, including LN229, U87MG, and LNZ308. Our results showed that only telmisartan decreased the viability of all three glioma cell lines in a dose- and time-dependent manner (Fig. 2A). The telmisartan IC50 values in LN229, U87MG, and LNZ308 calls after exposure for 48 h were 25.05 μM, 170.83 μM, and 318.13 μM, respectively. The MTS result of LN229 cells incubated with higher doses of telmisartan was shown in Additional file 1: Fig. S1. Moreover, 100ul Fimasartan reduced the viability of LN299 cells but not that of U87MG and LNZ308 cells (Fig. 2B), while valsartan did not affect any of the cell lines tested (Fig. 2C). Interestingly, LNZ308 cells, which showed the highest expression of AGTR1 (Fig. 1D), were not the most sensitive to ARBs. Rather, only telmisartan showed growth suppression effects on LNZ308 cells (Fig. 2A). These results suggest that the efficacy of ARBs as antiglioma agents may be due to pharmacological off-target effects.

### Telmisartan dose-dependently inhibits colony formation and proliferation of GBM cells.

**Fig. 3:**
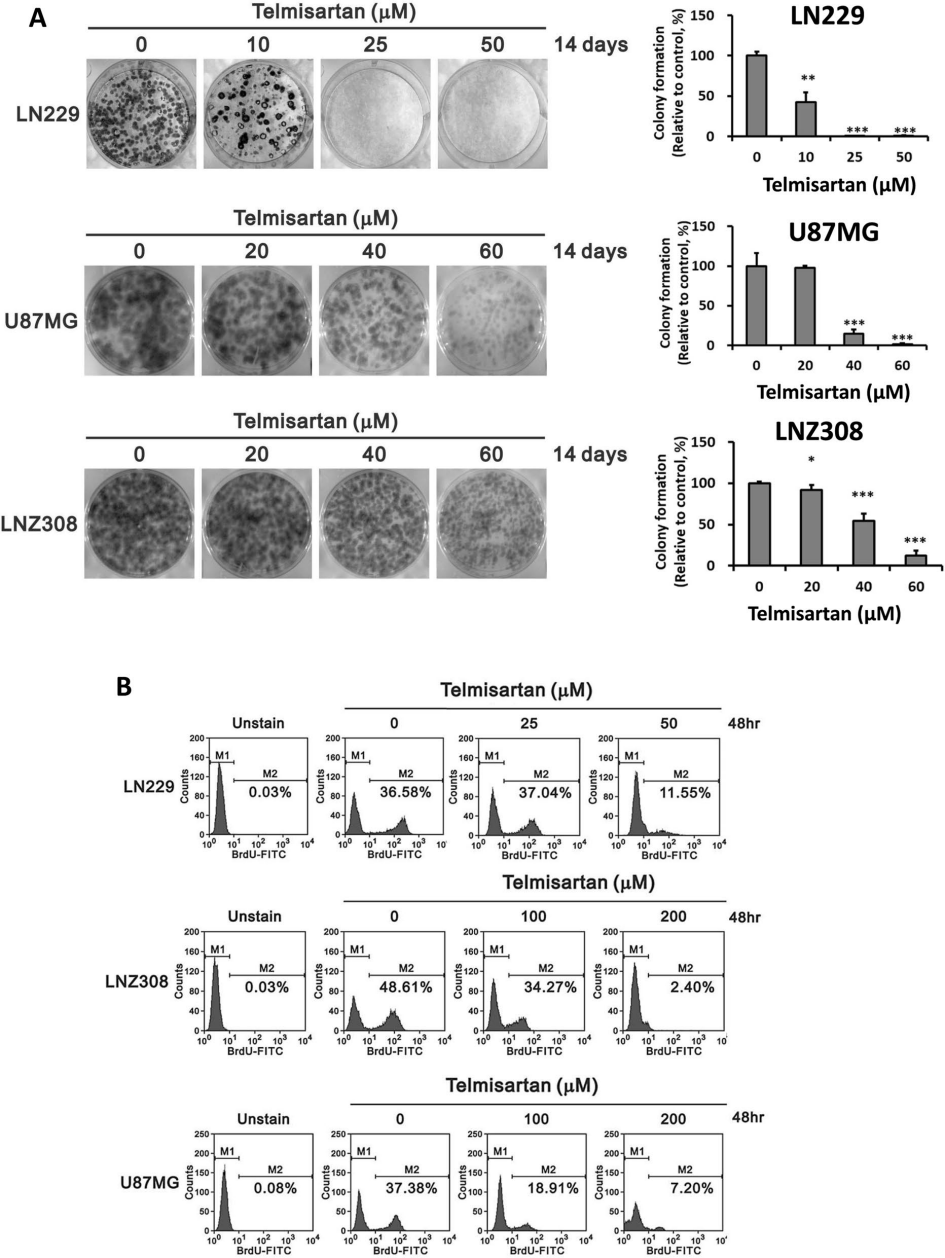
The effects of telmisartan on the colony formation and proliferation of human LN229, U87MG and LNZ308 glioblastoma cells. **A** Glioma cells were treated with DMSO or different concentrations of telmisartan for 14 days. The number of colonies is shown as the mean ± SD of 3 independent tests (** p < 0.01; *** p < 0.001). **B** Glioma cells were treated with telmisartan for 48 h, and then BrdU incorporation by cells was performed using a FITC BrdU Flow Kit. M1, BrdU-negative cells; M2, BrdU-positive cells. Cells untreated with BrdU were used as blanks. The results are representative data from two independent experiments. These results showed telmisartan dose-dependently inhibits colony formation and provokes apoptosis of GBM cells

To test the long-term antitumorigenic effect of telmisartan in GBM, we treated LN229, U87MG, and LNZ308 cell lines with telmisartan for colony formation assays. The results revealed that telmisartan dose-dependently repressed colony formation in three cell lines (Fig. 3A). We further examined the effect of telmisartan on cell proliferation. We observed that telmisartan deceased proliferation ability in a concentration-dependent manner in all 3 cell lines, as verified by BrdU flow cytometry analysis (Fig. 3B). The proportion of cells with BrdU staining among the untreated cells and the cells treated with the highest indicated dosage were 36.58% and 11.55% in LN229 cells, 37.38% and 7.20% in U87MG cells, and 48.61 and 2.40% in LNZ308 cells, respectively.

### ARB inhibits the migration and invasion of human LN229 and LNZ308 glioma cells

**Fig. 4:**
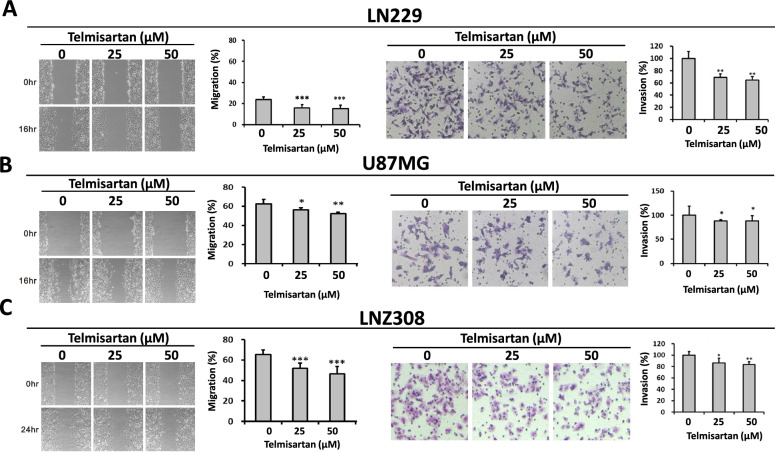
The effects of telmisartan on the migration of human LN229 and LNZ308 glioblastoma cells measured by wound healing assays and transwell invasion assays. **A**–**C** left, LN229, U87MG, and LNZ308 glioma cells were pretreated with mitomycin C overnight; the monolayers formed in 6-well plates were scratched, and the remaining cells were incubated with or without telmisartan for 16 h and 24 h. The groups treated with telmisartan for 48 h at the indicated dosage significantly had a significantly higher the wound area than the control groups in LN229, U87MG, and LNZ308 cells. The wound area was analyzed with ImageJ software and expressed relative to hour 0 (n ≥ 3; ***p < 0.001). **A**–**C **right, Representative photomicrographs of the underside of transwell filters demonstrating that compared to untreated cells, fewer glioma cells treated with telmisartan crossed the membrane from the upper chamber. These data demonstrated telmisartan suppressed the migration and invasion of human LN229, U87MG, and LNZ308 glioma cells

Aggressive invasion and migration are important phenotypic characteristics of GBM. Hence, we examined the effect of telmisartan on cell migration and invasion. The gap wound healing rate was significantly lower in the telmisartan-treated GBM cells than in the control cells (Fig. 4, left panel). Moreover, the number of invaded GBM cells in the telmisartan-treated group was significantly lower than that in the control group (Fig. 4, right panel). These findings verify that telmisartan suppresses the migration and invasion of human LN229, U87MG, and LNZ308 glioma cells at the indicated doses (Fig. 4A, B).

### Bioinformatics analysis of the gene expression profile of LN229 cells treated with telmisartan

**Fig. 5:**
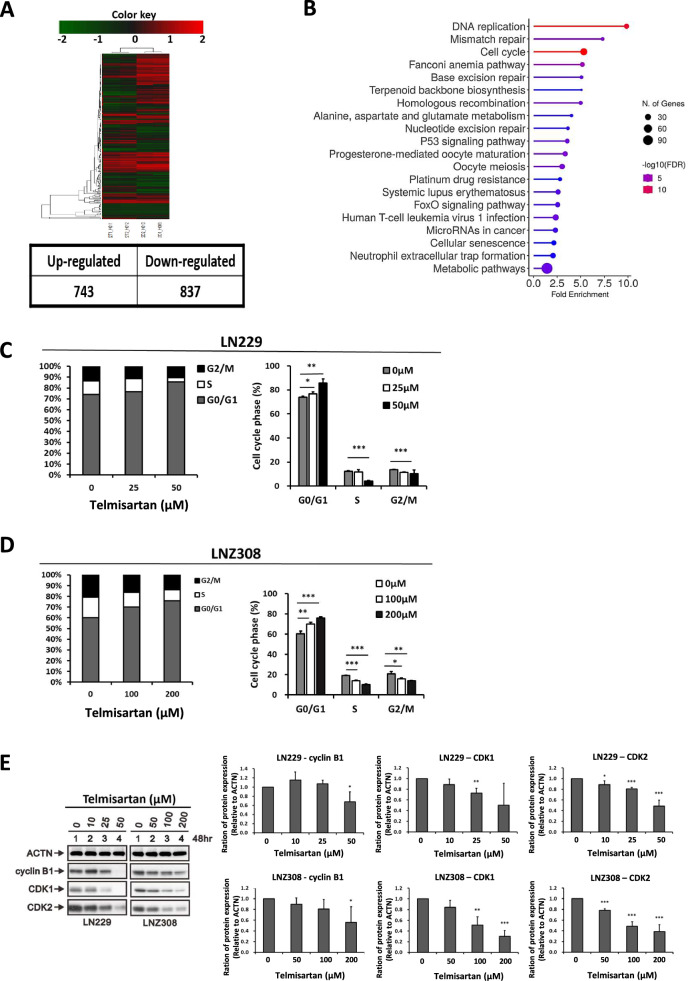
Microarray analysis revealed that telmisartan regulates DNA replication, mismatch repair, and the cell cycle pathway. **A** Representative microarray image of LN229 cells treated with telmisartan for 48 h. **B** Catalog of the top 20 signaling pathways changed by telmisartan treatment. The microarray data were analyzed using the KEGG database. **C**, **D** Cell cycle analysis by flow cytometric assessment based on PI staining of control and telmisartan-treated LN229, **C** GBM8401 and **D** LNZ308 cells. CellQuest software (Becton Dickinson) was used to determine the cell percentages in each phase (G0/G1, S, G2/M). The results are derived from three independent experiments. *p < 0.05, **p < 0.01, and ***p < 0.001 vs. controls by using the unpaired, two-tailed Student’s *t* test. **E** Levels of cell cycle-related proteins, including cyclin B1, CDK1, and CDK2, in LN229 and LNZ308 cells treated with the indicated dosage of telmisartan for 48 h. The relative expression of aforementioned proteins to ACTN was presented in the right panel (n = 3). *p < 0.05, **p < 0.01, and ***p < 0.001 vs. control cells. These data demonstrated telmisartan provoked GBM cell cycle arrest at the G1 phase

Since the abovementioned results demonstrated that telmisartan can be a potential antiglioma agent, we performed gene microarray analysis to reveal the putative downstream targets and pathways of telmisartan. Analysis of differentially expressed genes between samples with or without telmisartan treatment revealed 743 upregulated and 837 downregulated genes, which were visualized using a heatmap (Fig. 5A). We further found via KEGG pathway enrichment analysis that the antitumor effect of telmisartan may result from DNA repair mechanisms such as DNA replication, mismatch repair, and the cell cycle (Fig. 5B).

Since the cell cycle is the major pathway associated with telmisartan’s anti-GBM mechanism, we analyzed cell cycle profiles of LN229 cells after telmisartan treatment by PI staining and flow cytometry. There was a dose-dependent accumulation of cells in the G0/G1 phase in comparison with the untreated control (Fig. 5C, D). The proportion of LN229 cells in the G0/G1 phase was 74.1% in the untreated group and 85.6% in the 50 µM telmisartan group, and the proportion of LNZ308 cells in the G0/G1 phase was 60.4% in the untreated group and 76.1% in the 200 µM telmisartan group. To confirm these results, we performed western blotting of cell cycle regulatory proteins that are involved in the G1/S phase, including cyclins and CDKs (Fig. 5E). The results demonstrated that telmisartan decreased the levels of G1/S phase progression-related proteins, including cyclin B1, CDK1, and CDK2. In brief, telmisartan successfully provoked GBM cell cycle arrest at the G1 phase to reduce cell proliferation.

**Fig. 6:**
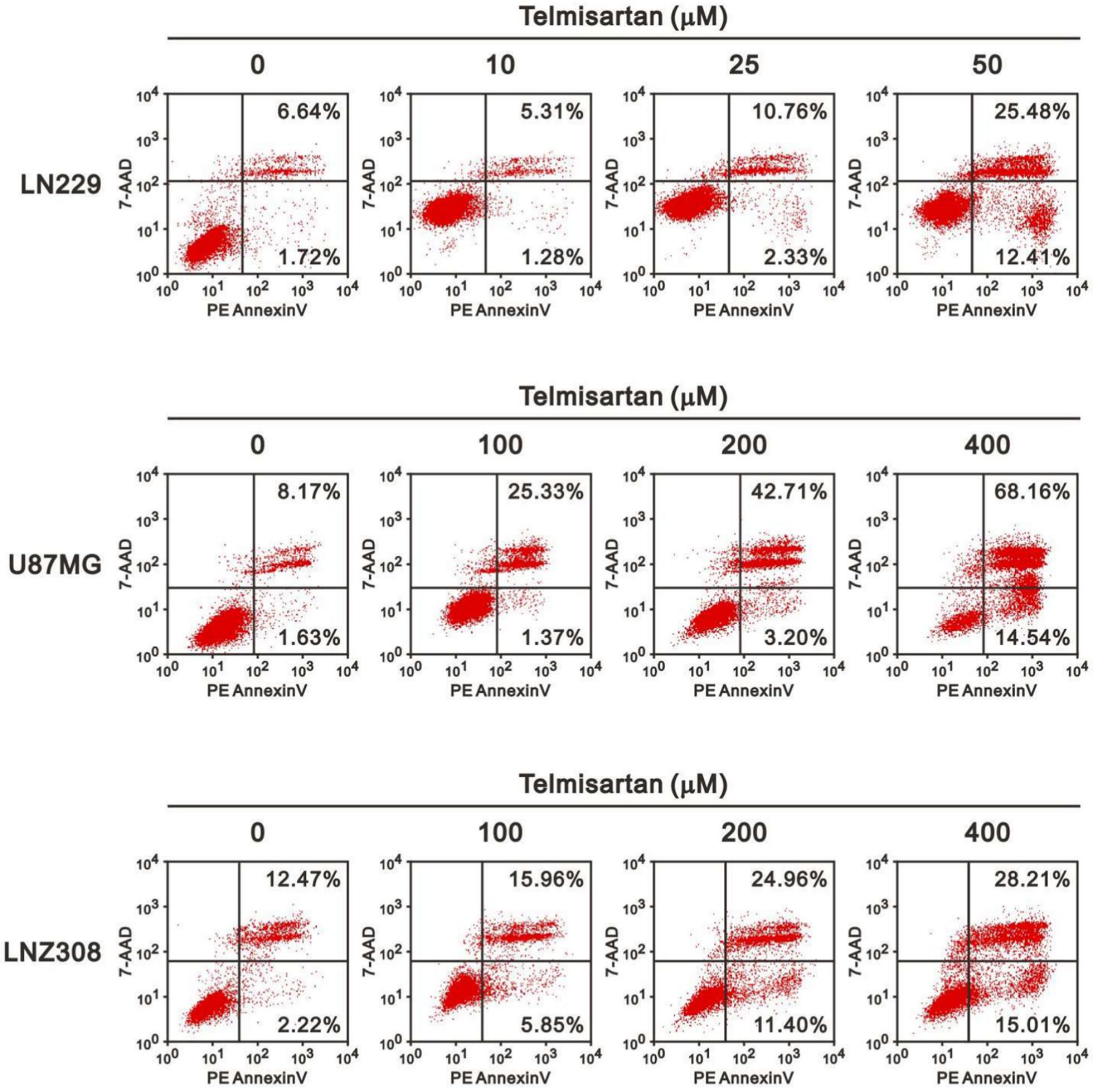
Telmisartan induced apoptosis in GBM cells. Effects of telmisartan on early and late apoptosis as demonstrated by flow cytometry based on annexin V and 7-AAD positivity. Treatment with the indicated dosage of telmisartan for 48 h stimulated apoptosis in almost all GBM cell lines. The x- and y-axes refer to annexin V and 7-AAD labeling, respectively. These results revealed telmisartan dose-dependently increased the proportion of apoptotic cells

Inhibition of the G1/S phase cell cycle transition suggests the possibility that GBM cells could either undergo DNA repair or apoptosis. Since telmisartan is highly likely to induce GBM cell apoptosis, we performed flow cytometry analysis of apoptosis by annexin V and 7-AAD staining to determine whether growth inhibition by telmisartan in GBM cells was associated with the induction of apoptotic cell death (Fig. 6). The percentages shown in Fig. 6 reveal a dose-dependent increase in the proportion of apoptotic cells. The proportions of apoptotic cells among the untreated cells and cells treated with the highest indicated dosage were 8.06% and 37.89% in LN229 cells, 9.8% and 82.7% in U87MG cells, and 14.69 and 33.22% in LNZ308 cells, respectively. The analysis of our microarray data using Gene Set Enrichment Analysis (GSEA) also observed that the HALLMARK_APOPTOSIS gene set was enriched (NES = 1.24 and FDR = 0.153) (Additional file 1: Fig. S2). This enrichment suggests that telmisartan may influence the expression of genes associated with apoptosis in glioma cells. In brief, telmisartan arrested the cell cycle at the G1 phase by provoking apoptosis.

### The bioinformatic analysis and western blotting results provide evidence that SOX9 is a downstream target of telmisartan

**Fig. 7:**
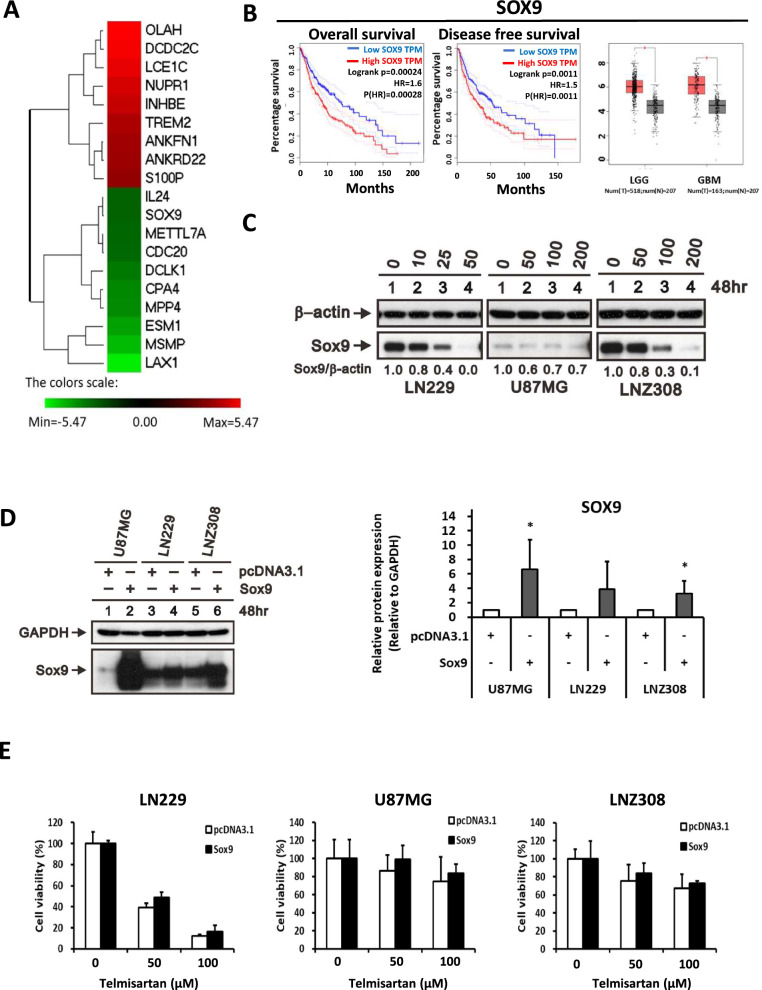
SOX9 is a downstream target of telmisartan. **A** The red and green bars represent the top ten most upregulated and downregulated genes, respectively. **B** left, Kaplan‒Meier overall survival and **B** middle, disease-free survival analyses revealed that the overexpression of SOX9 was associated with poor prognosis in glioma patients from the TCGA dataset. **B** right, SOX9 is overexpressed in LGG and GBM compared with normal controls. **C** Western blots showing levels of SOX9 induced by telmisartan in glioma cells. β-Actin was used as a loading control. **D** Glioma cells were transfected with the full-length human SOX9 plasmid. The overexpression of SOX9 was confirmed by western blotting. pcDNA3.1 was used as a transfection control. The quantification of abovementioned proteins relative to GAPDH was revealed in the right (n = 3). *p < 0.05. **E** Comparative MTS assay demonstrating the effect of SOX9 overexpression on LN229, U87MG, and LNZ308 cells treated with telmisartan at 48 h. Values are expressed relative to the control group (n = 3, error bars indicate ± SD). HR, hazard ratio; N, normal control; p(HR), pooled hazard ratio; T, tumor; TPM, transcripts per million; LGG, low-grade glioma. *p < 0.01. These data demonstrate SOX9 is a downstream target of telmisartan because SOX9 expression in glioma cells were dose-dependently inhibited by telmisartan. Moreover, GBM cells with Sox9 overexpression had a higher cell viability rate than control cells

To identify the downstream target involved in telmisartan’s antiglioma mechanism, we listed the top ten upregulated and downregulated genes of the LN229 microarray (Fig. 7A). Then, we analyzed the TCGA database. The outcomes revealed that high expression of SOX9 was associated with significantly shorter overall survival and disease-free survival times in TCGA glioma patients (Fig. 7B, C, left and middle panel). In addition, there was higher expression of SOX9 in GBM than in normal tissue (Fig. 7B, right panel).We further assessed whether SOX9 is the downstream target. As shown in Fig. 7C, telmisartan dose-dependently inhibited SOX9 expression in glioma cells. Moreover, LN229, U87MG, and LNZ308 cells overexpressing Sox9 (Fig. 7D) had a higher cell viability rate than control cells (Fig. 7E).

### Telmisartan inhibits tumor sphere formation and has an anti-GBM effect in vivo.

Previous studies have reported that sex-determining region Y (SRY)-box9 protein (SOX9) is an important transcription factor for neural stem cells during neurodevelopment [20]. Moreover, SOX9 also plays a critical role in stemness maintenance in glioma stem cells (GSCs) because silencing SOX9 suppresses GSC proliferation [21]. To further validate the anti-GBM effect of telmisartan on the self-renewal ability of tumor cells, we used GBM#1 and GBM#2 cells derived two fresh clinical patient specimens for the tumor sphere formation assay.

**Fig. 8:**
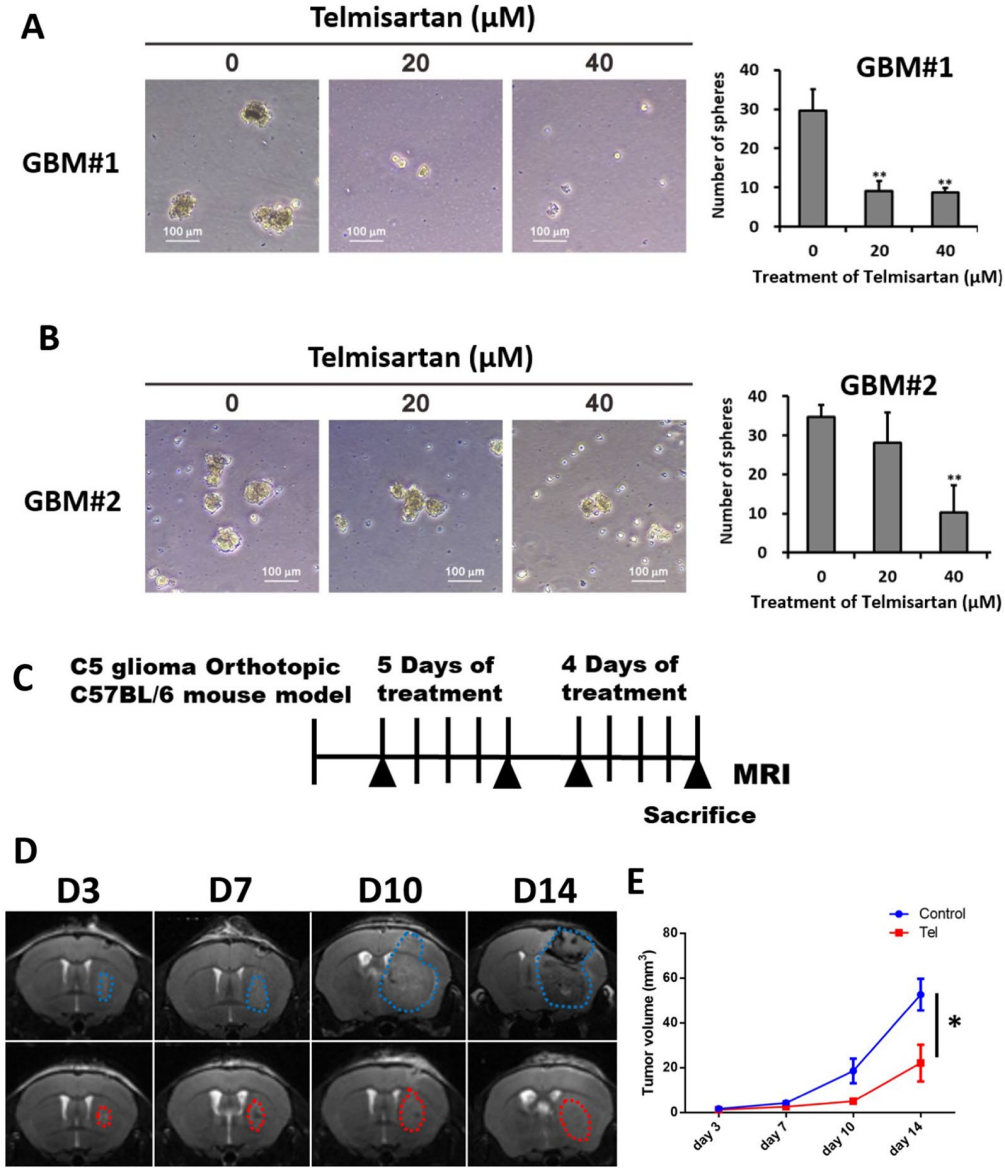
Telmisartan inhibits tumor sphere formation and has an anti-GBM effect in vivo. **A**, **B** Representative images of tumor spheres formed from 2 GBM patient specimens. Scale bar, 100 μm. The cells treated with the indicated dosage of telmisartan were cultured in 6-well plates for 14 days. Data are shown as the mean ± SEM. of at least four independent tests; **p < 0.01. **C** Treatment protocol for the C6 glioma orthotopic mouse model. **D** Representative MR images of mice on D3, D7, D10, and D14 after glioma cell implantation. **E** Time evolution of tumor volume on D3, D7, D10, and D14 after initial tumor inoculation and presented as SEM. n = 4 for each group. *P < 0.05. These data showed telmisartan inhibits tumor sphere formation and has an anti-GBM effect in vivo.

Representative images of primary GBM spheres after treatment with telmisartan for 14 days are shown in Fig. 8A, B. Telmisartan resulted in a visible decrease in the number of tumor spheres in comparison with that in the control group. Based on these results, telmisartan could inhibit the tumorigenicity of GBM cells.

To validate the anticancer effects of telmisartan in vivo, we used a C6 glioma orthotopic transplant mouse model. The mice were assigned to two groups: control and telmisartan (1 mg/kg). Telmisartan was administered intraperitoneally 4 days after C6 glioma grafting. The treatment protocol is shown in Fig. 8C. Brain tumor volume was estimated by MRI on D3, D7, D10, and D14. The brain MRI images demonstrated that administration of telmisartan (1 mg/kg) led to a significant decrease in tumor size (Fig. 8D, E).

## Discussion

The present study examined the association between *AGTR1* expression and survival time in glioma patients and evaluated the anti-GBM effect of three ARBs that can penetrate the blood–brain barrier. Only telmisartan exhibited a dose- and time-dependent antiproliferative effect in three GBM cell lines by inducing apoptosis and G0/G1-phase arrest evidenced by decreasing expression of cell cycle regulatory proteins. The arrest of the cell cycle transition at the G0/G1 phase providing cells an opportunity to undertake repair mechanisms or proceed through the apoptotic pathway. These outcomes are consistent with reported results of the antitumor effect of telmisartan on other cancers [22]. Furthermore, the antiproliferative effect of telmisartan was further validated in a C6 glioma orthotopic model.

Apoptosis has been shown to be a defense mechanism against cancer progression as it can eradicate mutated neoplastic cells from the system [23]. Apoptosis also acts as a major mechanism of cell death in a variety of cancer cells after cytotoxic drug treatment [24]. Our flow cytometry data suggest that treatment of GBM cells with telmisartan led to significant stimulation of apoptosis. These data were consistent with those of previous studies, which reported that telmisartan stimulated apoptosis in human endometrial [25], colon [26], and urological [27] cancer cells.

Accumulating studies have demonstrated that dysregulated RAS promotes malignant transformation in various cancers. Ma et al. reported that overexpression of *AGTR1* promotes the migration and invasion of breast cancer cells, which is associated with promoting lymph node metastasis. However, *AGTR1*-induced cell migration and invasion were inhibited by losartan, an AGTR1-specific inhibitor [28]. Zhang et al. reported that *AGTR1* expression was associated with poor epithelial ovarian cancer outcomes. *AGTR1* stimulation meaningfully increased the formation of multicellular ovarian cancer spheroid development, cell migration, and peritoneal metastasis [29]. Moreover, the metastatic ability of lung cancer was found to be correlated with angiotensin receptor expression, which could be significantly reduced by inhibition of RAS [30]. In this study, we validated that telmisartan significantly suppressed the migration and invasion of GBM cells.

SOX9 has been recognized for its oncogenic potency in several cancers. For example, Larsimont et al. reported that Sox9 is involved in the earliest step of tumorigenesis in basal cell carcinoma and regulates a specific gene network associated with tumor initiation and invasion [31]. Camaj et al. reported that knockdown of SOX9 expression in pancreatic tumor cells resulted in increased apoptosis and decreased migration in vitro and a significant decrease in primary tumor volume in an orthotopic injection animal model [32]. Wang et al. reported that SOX9 is an important factor for the maintenance of glioma stemness and gliosphere formation. In this paper, we found that SOX9 is downregulated in telmisartan-treated GBM cells. In addition, GBM cells overexpressing SOX9 had a higher cell viability rate than control cells under telmisartan treatment. Thus, our study identifies SOX9 as the potential downstream target of telmisartan in GBM treatment. This also explains the mechanism by which telmisartan inhibits the stemness of GBM cells since SOX9 is involved in gliosphere formation.

In clinical practice, angiotensin receptor blockers (ARBs) and angiotensin-converting enzyme (ACE) inhibitors (ACEIs) are commonly applied in the treatment of hypertension and other cardiovascular diseases. In the Asian population, ARBs are better tolerated than ACEIs because ARBs have fewer side effects, such as dry cough. Moreover, the use of ACEIs and ARBs to manage hypertensive cardiovascular disease in cancer patients is associated with better survival outcomes for breast, prostate, renal, and small cell lung cancer [33–36]. Kourilsky et al. [37] and Januel et al. [38] reported that GBM patients treated with Ang-II inhibitors had lower volumes of peritumoral edema and longer overall survival times than the non-ARB-treated group. In this study, we provided in vivo evidence that telmisartan suppresses GBM formation in a C6 glioma orthotopic model. Therefore, telmisartan has the potential to be a component of antiglioma combination treatment regimens.

## Conclusions

In this study, the molecular mechanism by which the AGTR1 blocker telmisartan contributes to GBM inhibition was clarified: it induces cell cycle arrest and provokes apoptosis. The results of this study provide a foundational understanding of the importance of AGTR1 inhibition in GBM growth and reveal the association between high levels of AGTR1 and poor clinical outcomes in GBM patients. This research also reveals a relationship between AGTR1 signaling and the SOX9 axis in gliosphere formation. Further studies are warranted to evaluate telmisartan’s potential as a component of anti-GBM combination strategies in vitro and in vivo.

## Supplementary Information

**Additional file 1: Figure S1.** Related to Fig. 2. Sensitivity of LN229 cells to telmisartan. LN229 cells were incubated with increasing doses of telmisartan measuring cell viability at 24, 47, and 72h. Values are expressed relative to those of the control group. This data reveals that LN229 cell viability is significantly decreased while treated with telmisartan greater than 100μM. **Figure S2.** Related to Fig. 6. The status of apoptotic genes in glioma cells under the influence of telmisartan. We performed an analysis of our microarray data using Gene Set Enrichment Analysisand observed that the HALLMARK_APOPTOSIS gene set was enriched. This enrichment suggests that telmisartan may influence the expression of genes associated with apoptosis in glioma cells. NES, normalized enrichment score; FDR, false discovery rate.

## Data Availability

The data that support the findings of this study are available on request from the corresponding author.

## Abbreviations

ARBs: Angiotensin II receptor blockers
GBM: Glioblastoma multiforme
CNS: Central nervous system
RAS: Renin-angiotensin system
AT1R: Angiotensin II receptor type I
AT2R: Angiotensin receptor type II
DMEM: Dulbecco’s Modified Eagle Medium
BrdU: Bromo-deoxyuridine
TCGA: The Cancer Genome Atlas
CGGA: Chinese Glioma Genome Atlas
ACE: Angiotensin-converting enzyme
TPM: Transcripts per million reads
