# Supplementary material for: Antiproliferative and apoptotic effects of telmisartan in human glioma cells
Source: Cancer Cell Int. 2023 Jun 9;23:111. doi: 10.1186/s12935-023-02963-1 (PMC10251602; doi:10.1186/s12935-023-02963-1)
Supplement: Supplementary file 1 — Additional file 1: Figure S1. Related to Fig. 2. Sensitivity of LN229 cells to telmisartan. LN229 cells were incubated with increasing doses of telmisartan measuring cell viability at 24, 47, and 72h. Values are expressed relative to those of the control group. This data reveals that LN229 cell viability is significantly decreased while treated with telmisartan greater than 100μM. Figure S2. Related to Fig. 6. The status of apoptotic genes in glioma cells under the influence of telmisartan. We performed an analysis of our microarray data using Gene Set Enrichment Analysisand observed that the HALLMARK_APOPTOSIS gene set was enriched. This enrichment suggests that telmisartan may influence the expression of genes associated with apoptosis in glioma cells. NES, normalized enrichment score; FDR, false discovery rate. [file 12935_2023_2963_MOESM1_ESM.docx]

**Cancer Cell International, Supplemental Information**

**Title: Antiproliferative and apoptotic effects of telmisartan in human glioma cells**

Yung-Lung Chang, Chung-Hsing Chou, Yao-Feng Li, Li-Chun Huang, Ying Kao, Dueng-Yuan Hueng, and Chia-Kuang Tsai*

**
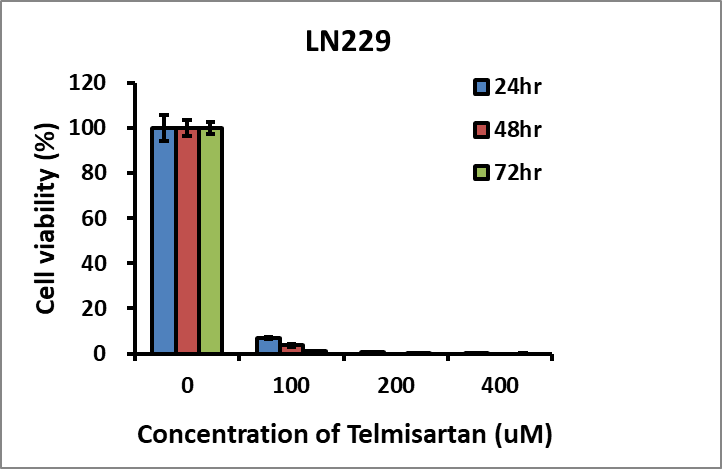
**

**Figure S1. Related to Figure 2. Sensitivity of LN229 cells to telmisartan.**

LN229 cells were incubated with increasing doses of telmisartan measuring cell viability at 24, 47, and 72h. Values are expressed relative to those of the control group (n=3, error bars indicate ± SD). This data reveals that LN229 cell viability is significantly decreased while treated with telmisartan greater than 100μM.


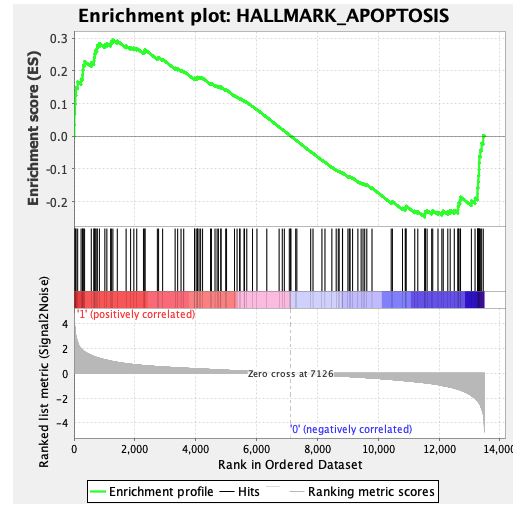


**Figure S2. Related to Figure 6. The status of apoptotic genes in glioma cells under the influence of telmisartan.**

We performed an analysis of our microarray data using Gene Set Enrichment Analysis (GSEA) and observed that the HALLMARK_APOPTOSIS gene set was enriched (NES = 1.24 and FDR = 0.153). This enrichment suggests that telmisartan may influence the expression of genes associated with apoptosis in glioma cells. NES, normalized enrichment score; FDR, false discovery rate.
